# On the Mechanism of Production of Symptoms of Diseases of the Brain

**Published:** 1873-08

**Authors:** C. E. Brown-Sequard

**Affiliations:** New York


### *On the Mechanism of Production of Symptoms of Diseases of the Brain.* By C. E. Brown-Sequard, M.D., New York.

It is my intention, in this first or introductory paper on diseases of the brain, to glance over the whole field of symptomatology relating to that organ, with the purpose of showing that we must give up as absolutely false most of our generally or universally admitted notions as regards the physiology and semeiology of the brain, and that new views must replace these old and effete notions. The advance of our knowledge in the symptomatology and the therapeutics of brain disease has long enough been barred by the generally accepted theories, which, as I will try to show, are completely erroneous. There is no important progress possible in the study of that subject, either for practice or science, unless we draw back and enter an entirely new path.

Since I began to study medicine, in 1838, the subject of this paper has constantly attracted my attention, and I selected it as the theme of the six Gulstonian Lectures I had the honor to deliver, in 1861, at the Royal College of Physicians of London. Since the last date I have made much progress in collecting arguments against the old and generally admitted views, and in building up the new theories I have to establish.

In this first paper I will merely give an outline of the different elements of my subject, but I shall take them up individually afterwards and study them at full length in as many papers or in a complete work. This paper will be divided into four parts.

### PART I.—VIEWS GENERALLY ADMITTED AS REGARDS THE ORIGIN OF SYMPTOMS OF BRAIN DISEASE.

I will first sum up briefly what is universally or generally admitted, and then give a short account of views which are accepted by a great many able men in this country and in Europe.

1. The nervous system is composed of two lateral halves, arranged so that the right side of the brain is the centre for the left side of the body, being for it the seat of the will and of the perception of sensations; while the left side of the brain is the centre for the right half of the body.

2. The conductors of the orders of the will to muscles coming from the brain, decussate, chiefly if not entirely, in the lower part of the medulla oblongata, so that those from the right half of the brain pass into the left side of the spinal cord, and *vice versa*.

3. The conductors of sensitive impressions from the whole right side of the body, in their way to the left side of the brain, and those from the left half of the body going to the right half of the brain, decussate, either entirely in the spinal cord and medulla oblongata, as I have tried to prove since 1849 (a view which I have been compelled recently to modify notably), or in those parts and in the pons Varolii, as many physicians, not knowing my arguments, yet admit.

4. One of the lateral halves of the brain is the seat of the perception of visual sensations, either for the whole eye on the other side (according to one view), or for the external half of the one eye and the internal half of the other (according to Wollaston's theory).

5. The vaso-motor nerves arise chiefly, if not entirely, from the medulla oblongata (Moritz Schiff, C. Ludwig, Soboroff, and others). They decussate partly in the spinal cord, partly in the medulla oblongata (M. Schiff).

6. The respiratory movements depend on conductors arising from the medulla oblongata, and descending without decussation along the spinal cord.

7. Certain limited parts of the brain have certain kinds of functional activity, as, for instance, the left third frontal convolution and neighboring parts, which are said to be the seat of the faculty of expressing ideas by speech,—the tubercula quadrigemina, which are considered as the centres for visual sensations, etc.

8. Symptoms such as diminution of intelligence, and the various disorders of that faculty in insanity; aphasia; loss of power of voluntary movements; anæsthesia; loss of the higher senses, etc., *are due in a direct way* to those alterations of tissue in the places where we find organic disease in the brain after death. In other words, these symptoms are explained by the assertion, *that the part diseased* (either by pressure or otherwise) *has lost its function.*

9. Delirium, convulsions, and some other active manifestations in cases of disease of the brain, *are due also in a direct way* to the irritation of the part of that organ where an organic alteration is found after death. In other words, the symptoms are explained by the assertion, *that the part diseased has had its vital property or properties morbidly put in play.*

I consider as erroneous not only the above generally admitted views, but also many others less generally received, but maintained by many able physiologists and physicians. Among these last opinions the following deserve notice: 1st. Todd and Bowman, and after them W. B. Carpenter, Broadbent, and others, have considered the corpora striata as centres for the voluntary movements, and the optic thalami as centres for sensations; 2d. Broadbent, in an excellent paper,* has propounded the view that one of the corpora striata is sufficient to act on the muscles of the trunk, neck, and some other parts on the two sides, explaining in that way the numerous cases in which these muscles escape paralysis in diseases of the brain; 3d. Various hypotheses have recently been put for-

* The British and Foreign Medico-Chirurgical Review. London, April, 1866, p. 468 et seq.

ward by Broadbent, Meynert, and Luys, to explain the mechanism of action of the brain in health and disease. The views of Meynert are ably expressed in an article by one of his pupils, Dr. James J. Putnam, of Boston, in this No. of these Archives; 4th. The efforts made by Nothnagel, Wilks, Meynert, J. Hughlings Jackson, and others, to localize the central cause of epilepsy and epileptiform convulsions in certain parts of the brain; 5th. The efforts of Duckworth, J. H. Jackson, and others, to localize the central cause of chorea in the corpora striata or the optic thalami; 6th. The efforts of R. Dunn, W. B. Carpenter, Ph. Lussana,* J. Hughes Bennett and others, to localize the muscular sense in the cerebellum.

I will not say more now on the views propounded or held by the ablest physiologists and physicians of our times on the general pathology of the brain; I will show hereafter how erroneous or incomplete they are.

PART II.—VIEWS WHICH I CONSIDER AS WELL ESTABLISHED OR MOST PROBABLE AS REGARDS THE PHYSIOLOGY AND THE PHYSIOLOGICAL PATHOLOGY OF THE BRAIN.

I will simply say now, that the following points are already well established or extremely probable, and in perfect harmony with all the facts I know.

1. The brain is a completely double organ, each of the hemispheres being a whole brain in itself, not only for mental actions as ably maintained by Sir Henry Holland,† and by Dr. A. L. Wigan,‡ but also for every other function (volition, intellectual perception of sensations, etc.) known to belong to the various parts of the central mass of nervous tissue named encephalon.

2. Originally no marked difference exists between the right and the left hemispheres, each of them being in newly-born children almost as well able by natural development and future exercise to acquire all the functional powers of the brain; but as one of the two hemispheres, being sufficient for certain functions, is exercised more than the other, it comes to be much more apt than the other to execute these functions. This view, ably maintained by Dr. W. Moxon,§ and accepted by my eminent friend Dr. P. Broca, so far as the faculty of speech is concerned, is just as well grounded, as I will show hereafter, for the power of volition on the muscles, and for several other functions of the brain.

3. The communications between muscles and the brain as centres for volition take place in a normal state by two (if not more)

* Journal de la Physiologie de l'Homme, etc. Vol. v, 1862, p. 427.

† Chapters on Mental Physiology. London, 2d Ed., 1858, Chap. VII, p. 179.

‡ A New View of Insanity.—The Duality of the Mind. London, 1844, *passim.*

§ British and Foreign Medico-Chirurg. Review. London, April, 1866, p. 481.

distinct sets of conductors, one passing from a lateral half of the brain to the muscles of the corresponding side in the trunk and limbs, and remaining in all their length in the same side of the cerebro-spinal axis; the other set (or sets) decussating with the homologous set, coming from the other side of the brain, and going to the muscles of the trunk and limbs on the opposite side.

4. The decussation of the conductors serving to voluntary movements takes place not entirely or even chiefly where most physiologists and physicians (including myself, up to some time ago) believe it to occur, *i. e.*, in the lower and front part of the medulla oblongata; but all along the median plane of the cerebro-spinal centre, from the crura cerebri to the lumbar extremity of the spinal cord, including, of course, the pons Varolii and the medulla oblongata.

5. The transmission of sensitive impressions, coming from one of the lateral halves of the body, takes place also by at least two sets of conductors, one going up to the brain in a direct way, *i. e.*, remaining in the corresponding half of the spinal cord and of the brain, while the other passes into the other half of these nervous centres, decussating all along the spinal cord and medulla oblongata with the homologous conductors coming from the opposite side.

6. The decussation of these last conductors takes place, as I have tried to prove since 1849, along the median plane of the spinal cord for the trunk and limbs, and of the encephalon for the nerves of general and special sensibility arising from that part of the nervous centres.

7. Very few conductors, direct or decussating, are sufficient for complete communication between the brain and the spinal cord, for sensibility and voluntary movements. These communications take place not by mere prolongations of the nerve-fibres of the motor and sensitive roots of the spinal nerves, after they have reached the cells of the spinal cord, but by a very small number of conductors, going from groups of these last cells to groups of brain-cells.

8. The vaso-motor nerves arise from many parts of the nervous centres (including the ganglions of the sympathetic). The medulla oblongata is the principal place of their decussation. The pons Varolii is the chief centre from which they originate, either for the viscera* or for the trunk and limbs.

9. The respiratory movements have their centre in the spinal cord, the pons Varolii, and other parts of the encephalon, as well as in the medulla oblongata. Ganglions in the diaphragm participate in the production of these movements.†

10. Symptoms of diseases of the brain are of two distinct

* See my paper on Hemorrhages due to a nervous irritation, in this Journal, p. 148.

† See the January No. of this Journal, p. 97.

classes: those which arise from an influence exerted on distant parts by an irritation of the diseased part (indirect symptoms), and those produced in a direct way, either by the loss of function or the irritation of the part diseased. The first class will be observed in all cases of diseases of the brain which produce symptoms. This first class will co-exist with the second in most cases of disease of the base of the brain; but it exists alone in cases of disease located in the cerebral lobes, the cerebellum or its peduncles.

11. The indirect symptoms are of two kinds, both arising, however, from an influence exerted on distant parts by an irritation starting from the organic lesion that we may see after death. Of the first kind are those symptoms due to a paralyzing or inhibitory influence; and of the second kind are those in which, instead of a cessation of activity, there is on the contrary the manifestation of a morbid activity.

12. To the group of indirect (inhibitory or arrestatory) symptoms belong the various kinds of cessation of activity which I have to study in the paper now in course of publication in this Journal (see January No., p. 88), including the loss of voluntary movements, anæsthesia, amaurosis, aphasia, loss of consciousness, loss of the power of controlling the sphincters, etc. I will show in that paper that all these kinds of cessation of activity take place in the same way as the arrest of the heart when the par vagum is galvanized.

13. To the group of indirect production of a morbid activity belong delirium, convulsions, and other active disorders of muscular movements.

14. The mechanism of production of the indirect symptoms of an organic disease of the brain seems to be identical with that of the reflected symptoms due to the irritation of peripheric nerves in the bowels, the lungs, the skin, or in other parts of the trunk and limbs. To be more complete, I ought to say that many nerve-fibres, either in the mucous membranes, the skin, the viscera, the trunks of the various spinal or cranial nerves, the cerebral meninges, the ventricular membranes, and the different parts of the brain itself, seem to have similar properties, and to produce indirect symptoms under an influence exerted by their irritation on distant parts.

15. In the same way that an irritation of a peripheric nerve will sometimes produce no indirect symptom (*i. e.*, a symptom due to an influence exerted on distant parts), or will in other instances produce any one of the indirect symptoms (those of cessation of activity or those of production of a morbid activity), in that same way will a lesion of the brain, extensive or not, produce no indirect symptom, or engender any one of the many indirect symptoms.

16. The above being admitted, it is easy to understand all the differences that may be found as regards symptoms of organic diseases of the brain. It is easy to understand how a paralysis can occur on the side of the brain lesion or on the opposite side; how a paralysis can appear suddenly and be complete although the lesion is small, and located in a part which cannot by any one be considered as the seat of the will, or containing the conductors for the voluntary movements of the part paralyzed; and how also a paralysis can disappear suddenly, although the brain lesion that gave rise to it still remains. It is easy to understand why there is no relation whatever between the extent, the kind, the seat and the rapidity of apparitions of an organic disease of, or an injury to, the brain, and the symptoms that may appear. I stop here, as I will have in the next part of this paper the opportunity to show that the old views are absolutely wrong, and that the theories I hold are in perfect harmony with the most important facts relating to the symptomatology of the organic affections of the brain.

In the next part of this paper I will also show that all the attempts heretofore made to localize certain functions or certain affections in special parts of the brain have, till now, been complete failures.

---

[*Second Article.*]

In the first article on this subject I have given the views universally or generally admitted concerning the origin of symptoms of brain disease, and also the views that I hold. I will, in this second section of my paper, give, first, the reasons that have led me to reject the old theories relating to this subject and to adopt the new views I propose, and then I will study the various modes of production of symptoms of disease of the brain. But before entering into the discussion of these important topics there are two points of great interest on which I have to say a few words.

(A.) I think the following statements may help the reader to understand more fully, and much more easily, the new views I try to establish :—

1. If we compare the symptoms produced by an irritation, such for instance as that of one of the lungs, or of the part of the base of the brain where the trigeminal nerve is inserted on the pons Varolii, or of the cerebral meninges, with the symptoms caused by an irritation of any part of the brain, we find that in either of the groups of cases we compare there may be paralysis produced in the corresponding or on the opposite side of the body, and, besides that, almost any of the symptoms of disease of the brain. On the other hand we find also, and in many cases, that no symptom of brain disease will appear, notwithstanding an organic disease in the cerebral lobes, in the base of the brain, in the cerebral meninges, or in the lungs.

2. Led by facts like the above, and by many others, I have necessarily come to the conclusion that there are nervous elements in the various parts of the cerebral lobes, in the corpora striata and optic thalami, in the crura cerebri and other parts of the base of the encephalon, which, like the nervous elements of the cerebral meninges, of the various viscera, the skin, the mucous membranes, the trunks of nerves, etc., have, when irritated, the power of producing on some part of the nervous centres—either at a short or at a very great distance from the seat of the irritation—changes in the normal state of activity of that part.

3. These changes may consist: *a*, in a *cessation of activity*, showing itself according to the part so affected by aphasia, agraphia, amnesia, paralysis, anæsthesia, amaurosis, anosmia, deafness, loss of taste, cardiac syncope, arrest of breathing, or a suspension of the interchange between tissues and blood; *b*, in the manifestation in a morbid form of the activity of certain parts of the nervous centres, producing delirium, convulsions, the so-called referred sensations, alterations of nutrition, etc.

4. In cases of alteration of the blood there is undoubtedly an influence of some poison on many parts of the brain and spinal cord, but there is also sometimes an irritation upon certain limited parts of the encephalon, propagated from them to other parts, just as in the case of a local organic disease, or just as in the case of an affection of the bowels, the lungs, the cerebral meninges, etc.*

5. From all the above statements, and from the study of every symptom of brain disease, I have drawn the conclusion that all the parts of the brain resemble the peripheric parts of the nervous system, in being able, under irritation, to act on any other of its parts, modifying their activity, so as to destroy, or diminish, or to increase and to morbidly alter it.

(B.) Independently of the old views which I criticise, and of the new views I try to establish, there are several well-demonstrated ways of explaining the production of symptoms in certain cases of brain disease. I will only say a few words about them here.

1. There is no doubt that alterations in the quantity or quality of the blood may cause symptoms of disease of the brain. I hardly need to name anæmia, congestion, the various kinds of toxæmia (uræmia, cholesteremia, oxaluria, etc.), among causes acting sometimes or generally on the whole mass of the brain. But local anæmia, local congestion, embolism, thrombosis (in an artery or a vein) act exactly like a wound, a hemorrhage, a tumor, as regards the mechanism of production of symptoms; and the criticism of the old views I will enter into hereafter, applies as well

* See the curious cases of *loss of speech caused by the bites of venomous snakes*, by Dr. William Ogle, in St. George's Hospital Reports, London. Vol. iii, 1868, p. 167.

to local changes in the amount of blood in the encephalon as to any local organic affection of that nervous centre.

2. A general pressure can, of course, destroy in a direct way the functions of the brain, but what I have just said about the amount of blood applies also to pressure. When it is localized it produces symptoms not according to the teachings of the old theories, but by the mechanism which I have pointed out for local organic affections of the brain.

3. There is no doubt that a considerable alteration of the whole extent of the cortical parts of the brain will affect the mental faculties in a direct way, *i. e.*, as taught by the old theories.

4. A propagation of organic alterations from the seat of an original lesion in the brain can, of course, give rise to symptoms. But we cannot explain the appearance of symptoms in that way in most cases of hemorrhage, of wound, or of softening of the brain, as it requires more time for the propagation of a morbid state to distant parts of the brain than there is between the moment the lesion takes place and of that apparition of symptoms. Even in cases of tumors enlarging slowly, we cannot look upon such a propagation as the essential cause of appearance of symptoms, as there are many cases of tumors in which there is no such propagation of a morbid state. R. B. Todd* considered that, when the surface of the brain was diseased, symptoms appeared only after the propagation by the cellular-tissue to the base of that organ. He only made a supposition that it was so. There are positive facts showing that a sudden lesion of the surface of the brain may at once cause symptoms (paralysis, loss of one or more senses, or convulsions, as well as mental disturbances). Then, of course, the symptoms cannot be due to an organic change extending to the base of the brain.

Another kind of propagation first discovered by L. Turck—studied since with great care by Prof. Charcot, by Bouchard, and others—consists in a peculiar degeneration, which progresses from the seat of a lesion in the corpus striatum or its neighborhood, down to the medulla oblongata on the same side, affecting in this last nervous centre the anterior pyramid, and with it passing into the lateral column on the other side of the spinal cord. Charcot and Bouchard have shown that even the nerves on the opposite side to that of the lesion in the brain may be degenerated. No doubt that in such cases the descending alteration must participate in the production of symptoms; but in these cases, also, the symptoms of brain disease appearing at once after a lesion of the encephalon cannot be due to this secondary alteration.

5. Two very able physicians, Prof. Vulpian† and Dr. Clifford

* Clinical Lectures on Paralysis, Disease of the Brain, etc. London, 1854, p. 49.

† Comptes Rendus de la Societe de Biologie, 1861, p. 38.

Allbutt, have had the idea that certain symptoms of brain disease may be due to an influence exerted by the diseased part of the encephalon on the secreting membrane of the lateral ventricles, producing an effusion of serosity. I will merely say, that although I agree with them in looking on such an influence as able to produce sometimes that effusion, there are two decisive reasons against giving to such a cause a great power. In the first place, we not rarely find a good deal of serosity in one or in both lateral ventricles without any symptoms; and in the second place, there are a great many cases of disease of those parts of the encephalon pointed out, by Prof. Vulpian and Dr. C. Allbutt, with such symptoms as they mention (amaurosis, paralysis on the side of the brain lesion, etc.), without any increase of the ventricular fluid.

### PART III.—SIGNIFICANCE OF THE GENERAL SYMPTOMATOLOGY OF BRAIN LESIONS.

Under this title I only intend to give a brief outline of the semeiology of the brain, with the double purpose of showing how completely it disagrees with the old or generally admitted views, and how much also it agrees with the new views mentioned in the preceding part of this paper. What I have to say on this subject, I will condense in a number of propositions, the demonstration of which I will try to give.

Proposition I.—*Lesions of a very small part of the brain often produce very marked symptoms.*—It is well known that lesions, limited to only a small part of the brain, will frequently produce symptoms. It would be easy to show that there is not one single small part, not only of the base of the brain, but of the three cerebral lobes—of their white or of their gray matter—which, being the seat of a lesion (hemorrhage, softening, or any other), has not sometimes produced marked symptoms, and, among others, paralysis. It is evident that the old theory of production of symptoms of brain disease is absolutely unable to explain such facts. The only way to get out of the difficulty is to suppose that the autopsy has not been well made, and that a lesion, not discovered, existed somewhere else than the one found, and that this supposed lesion was the cause of the symptoms. It is, of course, quite possible, if not certain, that real organic lesions have frequently escaped notice; but it would be a purely gratuitous supposition to admit that it always has been so in the very large number of cases in which only a small lesion has been found in the cerebral lobes in the bodies of persons who had had decided symptoms of brain disease. If we take the view I hold, that nerve-cells or nerve-fibres, or both, in the brain-tissue, like nerves of the bowels or other peripheric parts, possess, when irritated, the power to act on more or less distant parts of the nervous centres, and either morbidly put in play their vital properties, or stop or alter their functions, there is no difficulty to understand that any symptom of brain disease may arise from a lesion of a single circonvolution, or any other small part of the brain. As regards these two great points, *i. e.*, the lack of symptoms or the production of either one or another of the symptoms of brain disease, the history of cases of organic cerebral affections is similar to that of cases of worms in the bowels.

Prop. II.—*Extensive lesions may exist in the brain without marked symptoms.*—On the one hand, as above stated, a trifling lesion can produce decided symptoms, while on the other, extensive lesions may either exist without symptoms or produce only trifling effects. I will only mention here a few of such cases, and, in a special paper on this point, I will show that there is no part of the brain which has not sometimes been considerably altered or destroyed without the appearance of symptoms. Even the medulla oblongata, the pons Varolii, the crura cerebri, the corpora striata, the optic thalami, and the third left frontal convolution, have been found diseased in cases in which the symptoms (such as paralysis, anæsthesia, or aphasia), which ought to have shown themselves, had not, however, appeared.

Let us only study a few facts. Abercrombie,* whose accuracy cannot be denied, relates a case in which neither aphasia nor paralysis had existed, although almost the whole of the *left* hemisphere was considerably altered or destroyed. Here are his own words: "The brain externally appeared healthy; but when a thin section was cut from the upper part of the *left* hemisphere, a cavity was exposed, through which a probe passed in every direction without any resistance, through nearly the whole extent of the hemisphere. This arose from the whole hemisphere being in such a remarkable state of decomposition, or softening, that it formed one great cyst, full of soft, pultaceous matter, inclosed in a very thin covering, formed by the healthy cerebral matter on the surface. The healthy portion forming this covering in many places did not exceed a quarter of an inch in thickness, and at the thickest parts, which were on the upper surface of the brain, did not exceed one-half or three-fourths of an inch. The contained matter was a thin, soft pulp, mixed with portions of a pellucid, albuminous substance, which coagulated when thrown into boiling water. This matter was chiefly in irregular masses, but there were some finer portions of it which could be separated in the form of distinct round nodules, resembling hydatids. They were, however, only uniform masses of albuminous matter in a more concrete state. On the external part of the hemisphere, lying over the

* Pathol. and Practical Researches on Diseases of the Brain, etc. Edinburgh. 4th Edition, 1845. Pp. 176—178.

petrous portion of the temporal bone, there was a tumor the size of a pigeon's egg. The left ventricle was entire; it contained a small quantity of serous fluid, and was separated from the diseased mass by a very thin septum. The *left* hemisphere had been considerably enlarged, and the right diminished in the same proportion, the falx being sensibly pressed towards the right side."

In this decisive case all communication between the base of the brain and what remained of the superficial parts of the brain was cut off, and still there was no aphasia, no paralysis, no anæsthesia. For many years the brain had been diseased, as evidenced by various symptoms which had appeared and ceased (attacks of giddiness, of general rigidity, vomiting, amaurosis of the *left* eye, etc.). The patient was found dead one morning, after having been so well that she had spent the preceding evening with a party in the house of a friend.

As regards the part of the brain above the lateral ventricle, I will only mention one more case. It was observed by one of the ablest of the living surgeons of Paris, Prof. Richet. It is in some respects more important than Abercrombie's case. In another paper I will give it entirely. I will now simply say that an abscess of from 200 to 300 grammes (from seven to ten ounces) of greenish or milky pus had destroyed the middle lobe almost entirely, and a good part of the posterior lobe of the right hemisphere, and that the right lateral ventricle was filled with pus, and the right optic thalamus and corpus striatum were partly destroyed. Death occurred suddenly: the patient had had no paralysis, no anæsthesia, no convulsions, no intellectual deficiency.* For cases analogous to that one, I will refer to an excellent paper of my able friend, Dr. J. B. S. Jackson,† and to Dr. R. Bright's great clinical work.‡ In another paper, the publication of which I began in a preceding number of this Journal, on new kinds of hemiplegia, I gave several cases showing that a lesion of the base of the brain, instead of producing the ordinary crossed paralysis, may cause palsy in parts of the trunk and limbs where this loss of motor power should not be, according to the generally admitted theories.

I will only add that the medulla oblongata, the part of the nervous centres which is by far more able than any other to cause sudden death, when suddenly injured (and even very slightly sometimes), is also a part which can bear the greatest alteration without paralysis or anæsthesia in limbs.

Prop. III.—*There is no relation whatever between the extent of a lesion in the brain and the production of symptoms.*—On the one hand,

* See Alphonse Champsaur's thesis: De l'affection tuberculeuse du rocher. Paris, 1862, p. 30.

† See Boston Medical Journal, Vol. lxii, May, 1860, p. 353 et seq.

‡ Reports of Medical Cases. London, 1831, Vol. ii, pp. 151—165.

if we consider what has been said above, trifling lesions can produce decided symptoms; and, on the other hand, most extensive lesions, never mind where located, may produce neither paralysis nor anæsthesia, nor intellectual disturbances (aphasia, insanity, etc.). This can be expressed in other and perhaps more forcible words: *There is no relation whatever between the extent of a lesion in the brain and the symptoms that may be caused by it.* If symptoms were due, as is admitted, to the loss of function of the part altered or destroyed in the brain, or to *immediate* effects of the irritation of such a part, there would be a constant relation between them and the disease, so that the intensity and extent of the symptoms would be in proportion with the intensity and extent of the alteration.

PROP. IV.—*Symptoms of brain disease may exist without the least visible organic alteration of the brain.*—That paralysis, anæsthesia, or any other symptoms of disease of the brain may appear when that organ is without any organic disease, is a well-known fact, grounded on many cases in which a sudden or rapid cure was obtained after the cessation of a peripheric cause of irritation (the expulsion of worms, for instance), or in cases in which the autopsy has shown no alteration in the tissue of the brain. With the views I hold, this can be explained easily: the two kinds of symptoms (those of cessation of activity, *i. e.*, the inhibitory or paralyzing, etc., as well as those of irritation manifested by the morbid actions of parts of the brain (such as delirium, convulsions, etc.),—the passive and the active symptoms are due to the influence exerted by an irritation starting from a diseased organ upon certain parts of the brain, causing in some of them a cessation of activity, and in others, producing, on the contrary, a manifestation of activity.

The mechanism of production of symptoms of brain disease, as I have already said, and as I will, I hope, succeed to show in a special paper, is identically the same, whether the cause is in the bowels, or in any thoracic or abdominal viscus, or in the meninges of the brain, or in any part of the brain itself. The symptoms are not the *immediate* or *direct* effects of either the cessation of function or a morbid action of the part diseased. Wherever is the lesion which is the prime mover in the causation of symptoms, it entirely or at least partially produces them *mediately* or *secondarily*, or *indirectly*, and through the agency of an irritation starting from the seat of the lesion and acting on other parts which in a direct way give rise to the morbid manifestations.

PROP. V.—*The symptoms of brain disease may cease at the approach of death.*—This fact, which has attracted the attention of many able men, among others, Sir Henry Halford,* Guislain,† and Dr.

* Essays and orations read and delivered at the College of Physicians. 3d Edit. London. 1842. Pp. 17—20.

† Traite des Phrenopathies. 2d Ed., Bruxelles, 1835, p. 61.

R. La Roche,* cannot be explained by the generally admitted theory, while it is in perfect accord with my views. Not only just before death, but sometimes a little while before, symptoms of disease of the brain, depending on an organic disease of that nervous centre, will disappear more or less suddenly and completely. Admitting that they were produced indirectly, or mediately, or secondarily, and through the agency of an irritation starting from the seat of the lesion and acting on other parts, either to stop action or to generate it, we can easily understand that if the vital properties of the various parts of the brain (the part which is the seat of the lesion visible after death and also those parts acted on secondarily) are diminished in power from the diminution of circulation and hematosis which precedes death, it is natural that a cessation of the morbid manifestation of these properties should occur.

Prop. VI.—*A lesion of the brain can produce a cessation of symptoms of brain disease.*—There are facts on record showing that symptoms of brain disease may disappear after a considerable lesion of the brain. Thus in a case of insanity and epilepsy there was almost a complete cure after a fracture of the cranium and the issue of a notable quantity of brain tissue.† In another case there was improvement of the mental power after a lesion of the right side of the brain.‡ I know a case of a very intelligent gentleman, son of a most eminent statesman, who was naturally very reticent and who disliked to talk. After a fracture of the cranium just below the level of the left third frontal convolution, instead of losing speech, he came to be very fond of talking. Leaving this last case aside, the others, at any rate, cannot be explained by the generally admitted views, while they can easily be understood if we adopt the views I hold.

Prop. VII.—*An immense variety of symptoms in different individuals may be caused by a lesion in one and the same part of the brain.*—Symptoms of brain disease vary immensely, not only in intensity as stated already, but also in their kind, although the lesion producing them occupies the same place. This we witness most frequently. All the forms of convulsive affections, chorea, catalepsy, epilepsy, etc., all the varieties of mental disorder, all the differences in degree and location of paralysis, of anæsthesia and the various alterations of the senses, etc., have been observed in cases of disease of any one of the various parts of the base of the brain, and most of these symptoms have been observed in cases of disease of either the anterior, the middle, or the posterior lobes

* Journal of Psychological Medicine. New York. Vol. iii, p. 119 et seq.

† Gazette Medicale de Paris. 1844, p. 76; and another case, 1832, p. 95.

‡ Journal des Progres des Sciences et des Inst. Medec. Paris. Vol. xvii, p. 252.

of the brain. Are we to admit that each part of the brain possesses all the properties (normal or morbid) and all the functions of that great nervous centre? But if so, as we see that in some individuals there is no symptom at all, in others one symptom or another, we should have to conclude that one and the same part of the brain in some individuals is endowed with certain properties and functions, while in others it possesses entirely different properties and functions, and in others again, it does not seem to possess either a property or a function. The only rational explanation is, that in the same way that an irritation of any peripheric nerve* may either be insufficient to produce a remote effect, or quite able, on the contrary, to produce the most varied effects, a disease in any part of the brain may also be the starting-point of either an inefficient irritation or act, by means of that irritation on distant parts, and produce through them either kind of symptoms.

Prop. VIII.—*The same symptom may originate from the most various lesions.*—As regards the kind of lesions as well as concerning their extent and location, the most various organic alterations may produce the same symptom. Here, also, we can ask therefore: Are we to suppose that each part of the brain is endowed with the same vital properties and functions as all the others, and still more, as we know that what takes place for one symptom occurs also for every other, are we to suppose that every part of the brain can and does possess every property and every function of the whole brain? Or, are we to admit that in one individual a certain property or a certain function belongs to one part, in another individual to another part, and so on in as many individuals, to as many parts of the brain which produce the same symptom when organically altered?

Of course these suppositions are absurd, and as the old theories I am trying to prove false, could not, without the admission of these hypotheses, explain the fact that a lesion anywhere in the brain can produce the same symptom, we must give up these old theories. On the other hand, if we take up the view I hold, that symptoms in organic diseases of the brain arise in the same way as in cases of peripheric irritation (in the bowels, the lungs, etc.), we can easily understand that the irritation, starting from the place where we find disease after death, can go to any other part or set of parts of the nervous centres and produce the symptom that appears. Take amaurosis, for instance, and we find that it can be produced either in one or in both eyes, whatever be the seat or the kind of a lesion in the cerebral lobes, in the base of the brain, or in the cerebellum. If symptoms arose according to the old theories, we would have to place the centre for visual perceptions

* See my article on the remote consequences of nerve-lesions, in Holmes' System of Surgery. London. 1870. Vol. iv, p. 184.

everywhere. This is, of course, inacceptable, while if we admit that there are nervous elements, in the various parts of the encephalon, endowed—in different degrees in different individuals—with the power of arresting the activity of either the nerve-cells of the retina or of those which constitute the cėntre for visual perceptions, we can easily understand how, according to the degree of that power, there is or there is not, under the irritation caused by disease, amaurosis of one eye or both eyes, just as we see this deficiency of the sense of sight either in the two eyes or in one in cases of irritation of the bowels by worms.

PROP. IX.—*A lesion of the same kind and extent on the two sides of the middle line of the brain may produce symptoms only in one-half of the body.*—This is a most powerful argument against the old views I am criticising. There are a number of such cases on record. Abercrombie (*Loco cit*, p. 103) gives the case of a child, 16 months old, who had hemiplegia of the right arm and leg, and in whose brain there were, apparently, equal lesions on the two sides: several ounces of fluid in the ventricles and an abscess in the substance of the medulla oblongata which appeared to occupy "its whole diameter," where it is crossed by the pons Varolii. Huguier has reported a case of a tubercle, the size of a walnut, imbedded in the pons Varolii, appearing to extend as much on one as on the other side of the middle line, and having produced paralysis on the right side only (*Bulletins de la Societe Anatomique*, pp. 52–53, vol. iv, 1829). Proust relates a case of hemiplegia of the left side of the face and limbs, caused by syphilitic tumors placed over the middle line opposite the chiasma of the optic nerves.—(*Medecine eclairee par l'ouverture des Corps*. Paris, 1804, vol. ii, p. 59.)

I could relate several other well-observed cases, among others one of Abercrombie (p. 101), in which there was an abscess in each of the corpora striata with anæsthesia in only one side of the body; and another of Poisson, in which there was facial paralysis on one side, from a lesion of the pons Varolii by effused blood in the 4th ventricle (*Bull. de la Soc. Anat.*, 1855, vol. xxx, p. 204), but I think these facts are sufficient to show how untenable is the old view that paralysis, in organic affections of the brain, is due to the loss of function of the part altered. Here we see the same extent and kind of alterations in corresponding parts on the two sides of the middle line, in organs which are considered as containing the conductors for voluntary movements, and instead of the same amount of paralysis in the two sides of the body, we find hemiplegia. If we admit the view I hold, that the paralysis depends on the inhibitory or arresting influence exerted on other parts by an irritation starting from the seat of the lesion, it is easy to explain how there can be paralysis on one side only. As we know that irritations vary considerably in their power in different individuals, and in the same individual at different times, or in different parts of the body, or, besides, in corresponding parts of the two sides, we can easily understand that one side of the brain in the same individual will react powerfully under irritation, while the corresponding or rather the homologous parts on the other side will not react. Such a thing is not very frequent; but after all, if we consider that the nervous centres are double organs, a difference in the intensity of the excitability of one side, compared with that of the other side, is not more wonderful than the difference of the excitability of parts of the nervous system of one man compared with that of similar parts in another man.

PROP. X.—*Symptoms may appear indicating the existence of two lesions in the brain while only one exists.*—This has less value than the preceding proposition against the old view, because it can be said that another lesion existed which was not found. Still there are cases in which the nature of the lesion and the short duration of life after it had occurred, together with the known accuracy of the observer, leave very little doubt, if any, that symptoms which, according to the old theories, could only be produced by two distinct lesions, have been caused by one alone. In a case recorded by one of the late Presidents of the Royal College of Physicians of London, Dr. Thomas Mayo (*London Medical Gazette*, p. 16, vol. xxxix, 1847), a woman was stricken down by an effusion of blood in the *right* corpus striatum, and the autopsy showed no other lesion in the brain. The symptoms were: paralysis of the *left* limbs and of the *right* side of the face. My able friend, Dr. John W. Ogle, has seen just a similar case. The old theories cannot explain such cases except in admitting that although there was no lesion found elsewhere than in the right corpus striatum, there was one unseen causing the facial paralysis. If we admit my views, that paralysis in the face or limbs depend not on the loss of function of the part altered, but on an irritation of that part, and that an irritation on any part of the brain can produce paralysis either on the same side or the other, these cases can easily be explained. The patients in both of these cases had had an inhibitory arrest of the nerve-cells serving to the motion of the face on the side from which the irritation started, and an inhibitory arrest of the nerve-cells serving to the motion of the limbs on the other side.

PROP. XI.—*The two sides of the brain in adults react quite differently one from the other under the influence of irritations.*—This is in absolute opposition to the old theories about the physiology and symptomatology of the brain. As I will soon publish a special paper on this subject, I will only give now some of the conclusions arrived at by others or myself.

I will first speak of the left half of the brain, or rather, as it should be called, the left brain. I hardly need to say that aphasia and agraphia belong chiefly to the semeiology of the left brain. It is to Dr. Dax, Sr., that is due the merit of the discovery of this difference between the two brains,—the first difference pointed out between these two organs. But Dax committed the fault to consider this difference as absolutely constant. It is not so, as there are already on record (according to my own limited knowledge) more than thirteen cases of aphasia due to disease of the right brain with hemiplegia of the left limbs, and in one, at least, of these thirteen cases the patient was not left-handed.

Another symptom belongs also chiefly to the left brain: it is that a difficulty of articulation, or, in other words, a mechanical impairment of speech exists more frequently in affections of the left than in those of the right brain. This, to my surprise, although quite evident, has not been pointed out by any one except my former assistant, who has now risen so high, Dr. J. Hughlings Jackson.—(*London Hospital Reports*, Vol. ii, 1866, pp. 314–15.)

A third symptom belongs also more frequently to alterations of the left brain than to those of the right; it is insanity in its various forms. Acute delirium, however, seems to be more frequent in cases of disease of the right brain than in cases of disease of the left.

The right brain differs considerably from the left, not only by its being less frequently the cause of the three above-mentioned symptoms, but by its being more frequently diseased when the following symptoms exist:

1. Hysterical symptoms appear by far more frequently in the left than in the right limbs, which implies that when the brain is at fault in hysteria, the principal seat of trouble is in the right hemisphere. Canstatt and others had already pointed out that hyperæsthesia and spasm are more frequent in the right limbs, and anæsthesia and paralysis in the left limbs. It is known, also, that the pain under the breast exists oftener on the left than on the right side. Taking together the cases of unilateral paralysis in hysterical patients, mentioned by Macario, Briquet and De Fleury, besides the cases I have observed, I find that out of 121 cases, the left limbs were paralyzed in 97 cases and the right ones in 24 only. Two other very frequent differences exist in hysterical patients: there is a much greater frequency and a greater degree of tactile anæsthesia, with diminution of temperature due to spasm of blood-vessels, in the left than in the right limbs, particularly the hand.

2. Alterations of nutrition in paralyzed limbs are more frequent in cases of disease of the right than in cases of disease of the left brain. I have found that it is so for the so-called bed-sores and œdema. Out of 16 cases of bed-sores limited to one side, in the excellent paper of my friend, Prof. Charcot, proving that disease in one side of the brain produces sloughing in the opposite side of the body (*Archives de Physiol.*, Vol. i, 1868, p. 308), I find that the brain lesion was in the right side in 11 cases, and in the left in 5

cases only. I find that out of 19 cases of bed-sores mentioned by R. Bright, Andral, Abercrombie, and Lallemand, 13 existed when the lesion was in the right brain, and 6 when it existed in the left brain. Adding these facts to those of Charcot, we have 35 cases, in which 24 times the disease was in the right brain, while only 11 times it was in the left.

The alteration of nutrition in the ear of patients attacked with general palsy, is much more frequent in the left than in the right ear. (Petit, in *Journal de la Sect. de Medec. de la Loire Infer.*, Nos. 178–9. 1858, p. 299).

Optic neuritis, producing amaurosis, as my friend Dr. J. Hughlings Jackson has first ascertained, depends more often on disease of the right hemisphere than on disease of the left. (*Lond. Hosp. Rep.* 11, 1865, p. 316.)

Disease (hemorrhage, congestion, inflammation) of one or both lungs, due to disease in the encephalon, is more frequently found when the lesion is in the right than when it is in the left brain. Experiments on animals have shown this to me as well as clinical facts observed in man.

3. The convulsions of the muscles of the eye which constitute what Prof. Vulpian and Dr. Prevost have called the *conjugated deviation of the eyes* in organic brain affections, show that the right brain has much more power in producing that spasm than the left one. Adding a number of cases to those published by Dr. Prevost (*De la deviation conjuguee des yeux*, etc. Paris, 1868. *Passim*), I find that in 69 cases in which the convulsion was recorded by several authors or observed by myself, 47 times the lesion was in the right brain and 22 times only in the left.

I called attention long ago, in several lectures, to the greater frequency of general or unilateral convulsions in cases of lesions of the right brain than in those of the left. In very able papers of M. G. W. Callender (*St. Barthol.'s Hosp. Reports*, Vol. iii, 1867, p. 415, and Vol. v, 1869, p. 3, and *Med.-Chir. Transact.*, 1871, Vol. liv, p. 129,) I find that, without knowing my views, he has come to the same conclusions. As I will soon publish a paper on the subject of convulsions allied to or caused by diseases of the brain, I will only say here that, besides the general conclusion that diseases of the right hemisphere produce convulsions by far more frequently than diseases of the left one, I have ascertained the following interesting points: 1st, that diseases of the left brain, producing unilaterial convulsions, will very rarely indeed (in one out of ten cases) cause them in the same side; 2d, that diseases of the right brain, producing unilaterial convulsions, will, on the contrary, frequently cause them in the corresponding side (in more than one-third of the cases). So that taking together the cases of disease of the right or the left brain, unilateral convulsions have a greater tendency to occur in the right than in the left limbs.

4. We owe to Mr. Callender (*St. Barthol.'s Hosp. Rep.*, Vol. v, 1869, p. 24,) some remarks on the greater rapidity of death after apoplexy of the right hemisphere, as compared with that of the left. I have ascertained that his conclusion is borne out by the comparison of a great many more facts than those he has studied. I have also found that in softening of the brain by embolism, a similar result is obtained when we compare the right with the left brain. In 44 cases of embolism of the left cerebral arteries, the duration of life gives a total of 28,540 hours, which, divided by 44, gives an average duration of 648½ hours; while in 26 cases of embolism of the right cerebral arteries, the total being 13,110, the average duration has been 503⅘ hours.

Besides, I have made most careful experiments on a very large number of guinea-pigs, and obtained from injuries to one or the other of the hemispheres the following results: Out of 62 animals wounded in the right half of the head and brain, 14 only survived, *i. e.*, not one-fourth; while out of 34 with similar injuries to the left side, 19 survived, *i. e.*, more than one-half. To express myself perhaps in a more striking manner, death occurred after an injury to the right side in more than three-fourths of the animals, while it occurred in less than one-half of those whose left side was injured. I have unfortunately no exact record of the duration of life, but there is no doubt that this duration among the animals that died, was notably less for those operated on the right side than for the others. As much as possible the injuries were alike in the two sets of comparative experiments, and if there was a difference, it was that the right brain had been, perhaps, less injured than the left.

5. A very singular fact, pointed out long ago by Bayle and by Dechambre (*Bulletin Clinique*, Paris, 1835, p. 116), I have ascertained to be borne out by a great many cases of hemiplegia. It is, that when the unilateral paralysis is on the side of the lesion in the brain, it is almost always in the right hemisphere that this lesion is, or in other words, the right brain has much more power than the left to produce paralysis in the corresponding side of the body.

6. Another point of interest on this subject is, that the right brain is more capable than the left to produce also paralysis in the opposite side of the body. The frequency, the intensity and duration of hemiplegia are greater in the left limbs than in the right ones in cases of diseases of the opposite side of the encephalon.

7. A greater tendency to atrophy in cases of dementia exists for the right cerebral hemisphere than for the left, as shown by Baillarger, (*Annales Medico-Psychologiques*, 1858, Vol. iv, p. 168). Owing to that cause, left hemiplegia is more common than right side paralysis among the demented. The results obtained by

Baume (*Ann. Med. Psychol.*, 1862, p. 543), do not affect this conclusion (although they may seem to), because Baume has mixed up cases of hemorrhage and other morbid states with cases of atrophy.

I hardly need to say that all these facts, showing how different the symptomatology of the right brain is from that of the left brain, are absolutely irreconcilable with the old theories regarding the physiological and morbid actions and properties of the two sides of the brain. On the contrary, the views I hold are perfectly able to explain these facts. I will return to this subject in a special paper.

PROP. XII.—*In the same patient, with a persistent organic alteration of the brain, the symptoms may vary considerably from day to day.*—This variability is very often forcibly brought to the attention of medical men. Remarkable cases have been published by Casimir Broussais, Guislain, Abercrombie, Bright, Andral, and others. I will elsewhere give some of these cases. Such facts are usually explained by supposing that a congestion appears and disappears round the seat of the lesion. This supposition I will not deny *in toto*, as I admit the probability that such variable congestions have a good share in the causation of the symptoms. But can the old theories explain that a congestion (as was observed in a great many cases) seated in one limited part of the brain will produce alternately one set of symptoms and then another? Certainly not, while there is nothing contrary to the view I hold in the variability of symptoms from the same apparent cause. We know that a peripheric irritation may produce alternately various effects, and if we consider organic lesions of the brain as acting by a similar mechanism, it is quite natural that there be sometimes an alteration of effects.—*Archives of Scientific and Prac. Med.*

(TO BE CONCLUDED.)

